# Dietary encapsulated essential oils improve growth performance and regulate stress-responsive genes in heat-stressed broilers

**DOI:** 10.1093/jas/skaf417

**Published:** 2025-12-12

**Authors:** Mahmoud Madkour, Mohamed Hosny, Osama Aboelazab, Salem Fahmy, Gamal Rayan, Abdalla H H Ali, Yousef A Abdel Moati, Ahmed A Elolimy, Moataz Fathi

**Affiliations:** Animal Production Department, National Research Centre, Giza 12622, Egypt; Department of Animal Production, Faculty of Agriculture, Al-Azhar University, Assiut 71524, Egypt; Animal Production Department, National Research Centre, Giza 12622, Egypt; Department of Animal Production, Faculty of Agriculture, Al-Azhar University, Assiut 71524, Egypt; Department of Animal and Fish Production, College of Agricultural and Food Sciences, King Faisal University, Al-Ahsa 31982, Saudi Arabia; Department of Animal and Poultry Production, Faculty of Agriculture, South Valley University, Qena 83523, Egypt; Department of Animal Production, Faculty of Agriculture, Al-Azhar University, Assiut 71524, Egypt; Department of Integrative Agriculture, College of Agriculture and Veterinary Medicine, United Arab Emirates University, Abu Dhabi 15551, United Arab Emirates; Department of Animal and Poultry Production, College of Agriculture and Food, Qassim University, Al-Qassim 51452, Saudi Arabia

**Keywords:** antioxidant capacity, broilers, encapsulated essential oils, gene expression, heat stress

## Abstract

Despite increasing interest in the use of essential oils (EOs) as dietary supplements to alleviate the adverse effects of heat stress in poultry, the underlying biological mechanisms responsible for their protective actions remain incompletely understood. These mechanisms are likely influenced by a complex interplay of physiological responses, the specific bioactive constituents of the oils, and formulation variables such as encapsulation and dosage. This study evaluated the effects of dietary supplementation with a blended EO mixture, comprising cinnamon, thyme, and clove oils in a 50%:25%:25% ratio, and its encapsulated form (CEO) on growth performance, antioxidant status, and the expression of stress-responsive genes in broiler chickens exposed to natural summer heat stress conditions. A total of 350 one-day-old Cobb 500 male chicks were randomly assigned to seven treatment groups: a control group and six groups supplemented with EO or CEO at 50, 100, or 150 mg/kg diet. Each group consisted of five replicates with ten birds per replicate. The heat stress challenge was validated with a temperature-humidity index (THI) of 30.52, indicating moderate to severe thermal stress. Data were analyzed using one-way ANOVA, and treatment means were compared by Duncan’s multiple range test at *P* ≤ 0.05. Dietary supplementation with EO and CEO, particularly 100 mg/kg CEO (T5), significantly improved growth performance. At 42 d, T5 birds achieved the highest body weight (2380 g/bird; *P* < 0.01) and body weight gain (2336 g/bird; *P* < 0.01), feed conversion ratio was improved, with T5 showing the best FCR (1.45; *P* = 0.043) versus 1.56 in the control. Serum total antioxidant capacity (TAC) was highest in T5 (0.80 mM/L; *P* = 0.003), representing a 29% increase over the control. Hepatic expression of stress-responsive genes was markedly modulated. CEO at 100 mg/kg downregulated HSP70 by 58.1% (*P* < 0.001), HSP60 by 62.5% (*P* < 0.001), and HSPA9 by 62% (*P* < 0.001). Antioxidant enzymes SOD2, GPX1, and GPX4 were reduced by 75.2% (*P* < 0.001), 90.1% (*P* < 0.001), and 83.2% (*P* < 0.001), respectively. Pro-oxidant NOS2 and glucocorticoid receptor (GR) expression decreased by 50% (*P* < 0.05) and 55% (*P* < 0.05), respectively, while NRF2 and HMOX1 were downregulated by 51.5% (*P* < 0.05) and 62.7% (*P* < 0.05). Jejunal GLUT2 expression was upregulated by 227.3% (*P* < 0.05), whereas sGLT1 remained unchanged. No significant changes in liver enzyme activity or glucose levels were observed, suggesting that the treatments did not markedly impair hepatic function under heat-stress conditions (*P* > 0.05). These findings indicate that encapsulated thyme, cinnamon, and clove oils at 100 mg/kg can substantially enhance growth performance, antioxidant capacity, and cellular stress resilience in broiler chickens under heat stress.

## Teaser

Encapsulated cinnamon, thyme, and clove essential oils enhanced growth performance, strengthened antioxidant capacity, and downregulated hepatic heat shock protein expression in heat-stressed broilers. These findings support the use of encapsulated essential oils as a dietary intervention to help maintain productivity and bird welfare during periods of high environmental temperature.

## Graphical abstract

**Graphical Abstract:**
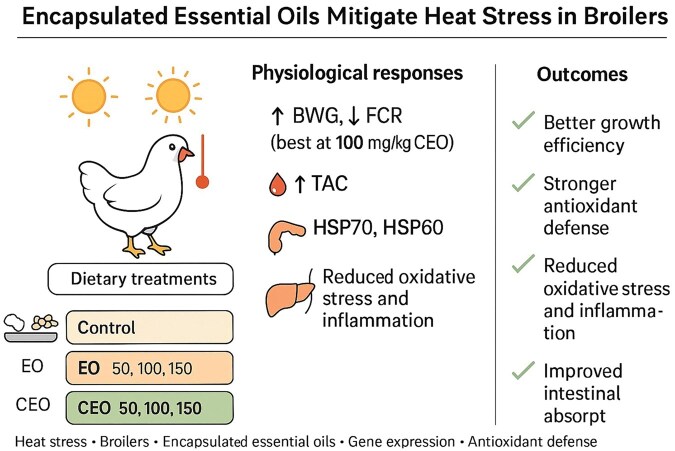

## Introduction

Heat stress is a serious issue affecting poultry farming worldwide. It negatively impacts the health, growth, immunity, and survival of birds, leading to substantial financial losses for the industry. (Madkour et al., 2021b; Oluwagbenga and Fraley, 2023). As climate change leads to hotter and more extreme heat waves, poultry farmers face growing challenges, especially with high-growth broiler breeds, which cause them to generate more body heat and make them particularly vulnerable to heat stress (Farghly et al., 2022; Oke et al., 2024).

Nutritional approaches are increasingly recognized as effective strategies to alleviate heat stress in poultry by enhancing antioxidant capacity, maintaining gut integrity, and supporting overall performance (Rhoads et al., 2013; El-Sayed et al., 2022; Al-Ghoul, 2023; Hashem et al., 2024; Madkour et al., 2025). In particular, essential oils (EOs) extracted from medicinal plants have gained significant interest. Their natural antioxidant, anti-inflammatory, and antimicrobial effects make them a promising solution (Hosny et al., 2020; Bao et al., 2023; Madkour et al., 2024a). These bioactive substances appear to help poultry adapt to stressful conditions by interacting with key stress-response systems, particularly those controlling heat shock protein levels (Shehata et al., 2020; Yilmaz and Gul, 2023; Madkour et al., 2024b).

Scientific studies demonstrate that cinnamon EO exhibits multiple beneficial effects, including antimicrobial and antioxidant capabilities, cholesterol-lowering properties, and digestive support through enhanced enzyme secretion (El Atki et al., 2019; Guo et al., 2024). Its main bioactive compounds, cinnamaldehyde, cinnamate, and cinnamic acid, have been linked to maintaining gut integrity and alleviating oxidative stress (Rao and Gan, 2014; Ali et al., 2021). Likewise, thyme EO, which contains high levels of thymol (∼40%) and carvacrol (∼15%), is recognized for its growth-promoting and health-supporting effects in poultry, including antioxidative, antimicrobial, and anti-inflammatory actions (Alkufeidy et al., 2022; Noruzi et al., 2022; Ali et al., 2024; Golshahi et al., 2025). Thymol, in particular, is associated with protective roles in metabolic, gastrointestinal, and neurological conditions (Nagoor Meeran et al., 2017; Vassiliou et al., 2023). Clove EO, rich in eugenol as its primary active constituent (typically comprising 70–85% of its composition, demonstrates a range of efficacy in poultry production. Its documented benefits include immunomodulatory and antimicrobial activities, which contribute to improved gut health and enhanced production performance (Haro-González et al., 2021; Suliman et al., 2021; Zhao et al., 2022). Thymol, eugenol, and carvacrol have closely related chemical structures, which may contribute to complementary or synergistic actions when combined. Such interactions can strengthen their biological effects and make them effective even at lower individual doses (Bassolé and Juliani, 2012). This synergy could amplify the antimicrobial, antioxidant, and growth-promoting effects of the blend, making it more effective than each compound on its own.

Based on this rationale, the present study utilized a blend of cinnamon, thyme, and clove EOs. Nevertheless, the application of EOs in practice is limited by their instability, low solubility in water, and vulnerability to environmental degradation (Cimino et al., 2021). To overcome these challenges, encapsulation techniques, such as spray drying, have been used to enhance the stability, bioavailability, and controlled release of EOs within the gastrointestinal tract (Favas et al., 2024; Babu et al., 2025). Indeed, the precise mechanisms by which dietary EOs mitigate chronic heat stress in poultry remain incompletely characterized and may be influenced by both the bird’s physiological responses and the way the oils are formulated.

This study aimed to investigate how dietary supplementation with a mixture of cinnamon, thyme, and clove EOs, provided either in free or encapsulated form, influences growth performance, blood biochemistry, expression of hepatic heat shock protein, antioxidant enzyme activity, and jejunal glucose transporter in broilers subjected to chronic heat stress.

## Materials and Methods

All experimental procedures used were approved by the Research Ethics Committee of the Faculty of Agriculture, Assiut University, Egypt, with reference no. 03-2025-0017.

### Essential oils analysis and microencapsulation

The three EOs used in this study were blended as follows: 50% cinnamon EO, 25% thyme EO, and 25% clove EO (0.5:0.25:0.25). Gas chromatography–mass spectrometry (GC–MS) analysis identified the primary compounds of the blended EOs as follows: Cinnamon oil contained 83.6% cinnamaldehyde; thyme oil consisted of 48% thymol, 17.5% γ-terpinene, 13.6% p-cymene, and 11% carvacrol; and clove oil contained 84% eugenol and 7.8% eugenol acetate. The microencapsulation of the blended oil was carried out using spray-drying technique as stated by (Gharsallaoui et al., 2007).

### Feeds and birds’ management

Seven experimental groups, each consisting of 50 one-day-old broiler chicks (Cobb 500), were established from the 350 male chicks used in this study. Each treatment group consisted of five replicates, with ten chicks allocated to each replicate. The control group received only the basal diet, whereas the second, third, and fourth groups were provided with the basal diet supplemented with a blended EO mixture at inclusion levels of 50, 100, and 150 mg/kg, respectively. The fifth, sixth, and seventh groups were given the basal diet enriched with. 50, 100, and 150 mg encapsulated blend of the EOs (CEO)/kg diet, respectively. The experimental period lasted six weeks.

The basal diet covered the nutritional requirements of broiler chicken according to the guidebook of Cobb 500 broiler. During the experiment, there was free access to drinking water and feeds were offered ad libitum. For the first 21 days of the trial, the chicks were given the starter diet; for the remaining days, they were fed the grower diet (Table 1). Environmental, hygienic, and managerial conditions were recorded for each bird.

**Table 1.** Formulation and nutrient composition of the basal diets

Ingredients	Starter diet (%)	Grower diet (%)
**Corn (maize)**	58	60.68
**Soybean meal, 46%**	28.1	26.9
**Corn gluten meal**	8.58	5.9
**Sunflower oil**	1.2	2.8
**Di-calcium phosphate**	2.3	2.0
**Limestone**	1.0	0.9
**Premix^1^**	0.30	0.30
**NaCl**	0.30	0.30
**L-lysine-HCl**	0.15	0.15
**DL-Methionine**	0.07	0.07
**Total**	**100**	**100**
**Calculated analysis^2^ (as feed basis)**		
**Metabolizable energy (kcal/kg)**	3004	3108
**Crude protein, %**	23.01	21.05
**Crude fiber, %**	3.44	3.38
**Calcium, %**	1.05	0.93
**Available phosphorus, %**	0.51	0.44
**Lysine, %**	1.20	1.14
**Methionine, %**	0.53	0.48
**Methionine+ cystine, %**	0.85	0.75

1 Vitamin premix supplied per Kg of diet: Vit A, 12000 IU; Vit D_3_, 2200 IU; Vit E, 10 mg; Vit K_3_, 2 mg; Vit B_1_, 1 mg; Vit B_2_, 4 mg; Vit B_6_, 1.5 mg; Vit B_12,_ 10 mg; Niacin, 20 mg; Pantothenic acid, 10 mg; Folic acid, 1 mg; Biotin, 50 mg; phytase enzyme and xylanase enzyme.

2 According to NRC (1994).

### Temperature–humidity index

The experiment was conducted during the summer season. The ambient temperature and relative humidity were measured twice daily during the experimental period using digital hygrometers. The following formula, as reported by Marai et al. (2001), was then used to calculate the Temperature–humidity index (THI):


THI = db °C − [(0.31 − 0.31RH)(db °C − 14.4)]

where db °C = dry bulb temperature in degrees Celsius and RH = relative humidity/100.

**Figure 1.**
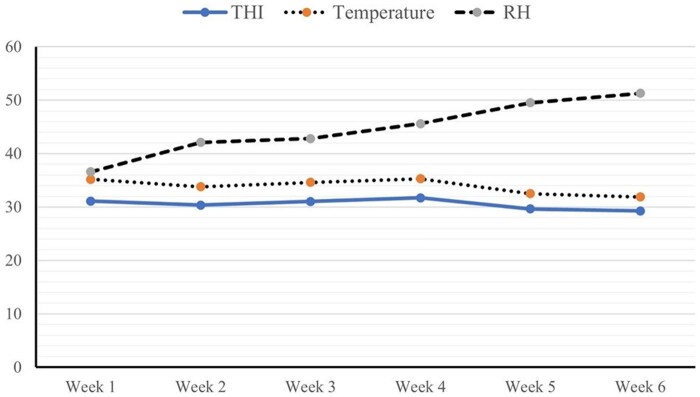
Ambient temperatures (°C), relative humidity (%), and temperature–humidity index (THI) during the experimental period.

The recorded environmental data indicated that the average ambient temperature and relative humidity during the trial were 33.9°C and 44.7%, respectively. The THI scores were categorized into the following classes: THI < 27.8 = no heat stress, 27.8 < THI < 28.9 = moderate heat stress, 28.9 < THI < 30.0 = severe heat stress, and THI ≥ 30.0 = very severe heat stress. Based on these values, the calculated temperature-humidity index (THI) was 30.52, confirming that the broilers were subjected to heat stress throughout the experimental period Tao and Xin (2003) (Figure 1).

### Growth performance parameters

Live body weight and weight gain were recorded on a weekly basis throughout the experiment. Although weekly measurements were collected, the data are presented as summarized intervals to align with standard broiler production phases: the starter phase (weeks 1–3), the grower phase (weeks 3–6), and the overall period (weeks 1–6). Therefore, the reported values represent cumulative performance for these intervals rather than individual weekly records. Feed intake and feed conversion ratio (FCR) were recorded following the same intervals, with FCR calculated as the ratio of feed consumed to weight gained (g feed/g gain).

### Blood biochemistry

At the end of the trial, five birds from each treatment group were randomly selected and manually slaughtered. Birds were humanely euthanized by cervical dislocation, followed immediately by exsanguination via the jugular vein. Blood was drawn at the time of slaughter using sterile tubes for subsequent analysis. After 20 min, the clot had fully formed, allowing clear serum to separate by centrifugation at 3500 rpm (2328.24 G)/15 min. Serum samples were stored at −20°C till biochemistry analysis (El-Wardany et al., 2016; Alagawany et al., 2022; Reda et al., 2022). Serum glucose, AST, ALT, MDA, and total antioxidant capacity (TAC) were measured using commercial kits (Bio-diagnostic Co., Egypt) following the manufacturer’s instructions. AST and ALT activities were determined colorimetrically based on the formation of 2,4-dinitrophenylhydrazone derivatives from oxaloacetate or pyruvate, respectively. MDA was measured using the thiobarbituric acid (TBA) method, forming a pink adduct read at 534 nm. TAC was assessed by the ability of serum antioxidants to neutralize exogenous H_2_O_2_, with the residual H_2_O_2_ quantified colorimetrically via enzymatic conversion to a colored product.

### Total RNA isolation and real-time quantitative RT-PCR

Upon slaughtering, jejunal and liver tissues were rapidly dissected, immediately frozen in liquid nitrogen, and stored at −80°C until further gene expression analyses. Approximately 20 mg of jejunal or frozen liver tissue were promptly immersed in TRIZOL reagent (Invitrogen Life Technologies, Palo Alto, CA, USA) for total RNA extraction, as per the manufacturer’s guidelines (Hemida et al., 2023). RNA purification was carried out using the RNeasy Mini Kit (Qiagen, Valencia, CA, USA). The concentration and integrity of the RNA were assessed using a NanoDrop 1000 spectrophotometer (NanoDrop Technologies, Rockland, DE, USA) and validated via agarose gel electrophoresis (Thermo Scientific, Wilmington, DE, USA). The Complementary DNA (cDNA) Synthesis Kit (Thermo Fisher Scientific, Catalog No. K1621) was utilized for cDNA synthesis.

Conventional PCR was first performed to identify the optimal annealing temperatures for each primer set (Table 2) and to ensure specificity, with products verified on 2% agarose gels. Quantitative real-time PCR (qPCR) was then carried out using a QuantStudio 5 system (Applied Biosystems). Each 15 µL reaction contained 7.5 µL of 2X SYBR Green Master Mix (Thermo Fisher Scientific, Cat. #K0251), 0.3 µM of each primer, 2 µL of cDNA, and nuclease-free water. β-actin served as the internal control. All reactions were run in duplicate, with mean Ct values calculated. Relative gene expression was normalized to β-actin and quantified using the 2^(−ΔΔCt)^ method.

**Table 2.** A list of the primer sequences and their corresponding annealing temperatures used in this study

Gene	**Primer sequence (5**′**-3**′**)**		Reference sequence	Annealing Tm (°C)
**HSP60**	F	CGCAGACATGCTCCGTTTG	NM_001012916	56
	R	TCTGGACACCGGCCTGAT		
**HSP70**	F	GATCTGGGCACCACGTATTCT	FJ217667.1	58
	R	GGTTCATTGCCACTTGGTTCTT		
**HSP90A**	F	ACA CAT GCC AAC CGC ATT TA	NM_001109785.1	57
	R	CCT CCT CAG CAG CAG TAT CA		
**HSPA9**	F	GGC ATG ACC AGA ATG CCA AAG	NM_001006147.1	56
	R	TTG TGA AGA CAC CGC CTA ATG T		
**SOD**	F	AGGGGGTCATCCACTTCC	NM_205064.2	54
	R	CCCATTTGTGTTGTCTCCAA		
**SOD2**	F	CTGACCTGCCTTACGACTATG	NM_204211.2	56
	R	CGCCTCTTTGTATTTCTCCTCT		
**GPX1**	F	TTCGAGAAGTTCCTCGTGGG	NM_0012778553.3	58
	R	CCTGCAGTTTGATGGTCTCG		
**GPX4**	F	GGGGAATGCCATCAAGTGGAA	NM_204220.3	57
	R	TAGGCGGGCAGATCCTTCTC		
**HMOX1**	F	AGGGTTGGCTTTCTTCACCTT	NM_205344.2	55.5
	R	TCCTGCTTGTCCTCTCACTCT		
**NFE2l2**	F	GTGACCCAGTCTTCATTTCT	XM_046921130.1	53
	R	GCTCGTGATTGTGCTTACTT		
**NOS2**	F	ACTGAAGGTGGCTATTGGGC	NM_204961.2	55
	R	TGTTTGTCTCCTTCCGCTGT		
**NOX4**	F	CCAGACCAACTTAGAGGAACAC	NM_001101829.3	55
	R	TCTGGGAAAGGCTCAGTAGTA		
**GR**	F	GCCATCGTGAAAAGAGAAGG	NM_001037826.1	54
	R	TTTCAACCACATCGTGCAT		
**SGLT or SLC5A1**	F	CAGAACGTTTGAGGGCTTTGT	NM_001293240	55
	R	AGCAAGTGGAGCCAATCAGA		
**GLUT2 or SLC2A2**	F	CACACTATGGGCGCATGCT	NM_207178.2	57
	R	ATTGTCCCTGGAGGTGTTGGTG		
**β-actin**	F	CACAATGTACCCTGGCATTG	L08165.1	54–56
	R	ACATCTGCTGGAAGGTGGAC		

### Statistical analysis

One-way analysis of variance was applied to the data. The General Linear Model of SAS (1994) for PC was used, and Duncan’s multiple range test (Duncan, 1955)was used to separate the significant differences between treatment means at the 5% probability level.

## Results

The study investigated the impact of varying doses of blended EO and encapsulated blended EO (CEO) on the performance of broilers exposed to heat-stress conditions. The blended EO was composed of cinnamon oil, thyme oil, and clove oil in a 0.5:0.25:0.25 ratio. Gas chromatography–mass spectrometry (GC–MS) analysis revealed the major active compounds as follows: cinnamon oil contained 83.6% cinnamaldehyde; thyme oil consisted of 48% thymol, 17.5% γ-terpinene, 13.6% p-cymene, and 11% carvacrol; and clove oil contained 84% eugenol and 7.8% eugenol acetate.

### Effect of essential oils (EO) and encapsulated essential oils (CEO) on the growth performance of heat-stressed broilers

As shown in Table 3, body weight (BW) was significantly influenced by the dietary treatments (*P* < 0.01). At 21 days of age, birds in T5 (100 mg/kg CEOs; CEO100) exhibited the highest BW (750 g/bird), followed by T2 (100 mg/kg EOs; EO100), T1 (50 mg/kg EO), and T3 (150 mg/kg EO). The Control, T4 (50 mg/kg CEO), and T6 (150 mg/kg CEO) groups recorded comparably lower BW values.

**Table 3.** Effects of dietary essential oils and their encapsulated forms on growth performance in heat-stressed broiler chickens

Trait	Experimental groups							MSE	*P*-value
	Control	T1	T2	T3	T4	T5	T6		
**Body weight, g/bird**									
**Age (days)**									
**1**	44	44	44	44	44	44	44	0.36	0.516
**21**	655^c^	716^b^	721^b^	706^b^	657^c^	750^a^	660^c^	15.1	<.010
**42**	2146^d^	2212^c^	2288^b^	2176^d^	2158^d^	2380^a^	2218^c^	41.4	<.010
**Body weight gain, g/bird/period**									
**Period (days)**									
**1–21**	611^c^	672^b^	677^b^	662^b^	613^c^	706^a^	616^c^	15.03	<.010
**21–42**	1491^c^	1496^c^	1567^b^	1470^c^	1501^c^	1630^a^	1558^b^	40.28	<.010
**1–42**	2102^d^	2168^c^	2244^b^	2132^c^	2114^d^	2336^a^	2174^c^	41.47	<.010
**Feed intake, g/bird/period**									
**Period (days)**									
**1–21**	975	1009	972	1040	963	1056	981	51.3	0.055
**21–42**	2297	2235	2300	2222	2195	2385	2186	127.5	0.200
**1–42**	3271	3244	3272	3262	3159	3441	3167	145.8	0.092
**Feed conversion ratio, g feed intake/g weight gain**									
**Period (days)**									
**1–21**	1.59^a^	1.50^b^	1.43^c^	1.57^a^	1.57^a^	1.50^b^	1.59^a^	0.07	<.010
**21–42**	1.54^a^	1.49^ab^	1.47^b^	1.51^a^	1.46^b^	1.47^b^	1.40^c^	0.07	0.049
**1–42**	1.56^a^	1.49^ab^	1.46^b^	1.53^a^	1.49^ab^	1.47^b^	1.45^b^	0.06	0.043

a,b,c,d Means within the same row with no common superscript differ significantly.

T1: 50 mg/kg essential oil; T2: 100 mg/kg essential oil; T3: 150 mg/kg essential oil; T4: 50 mg/kg capsulated essential oil; T5: 100 mg/kg capsulated essential oil; T6: 150 mg/kg capsulated essential oil. MSE: mean square error. Statistical significance among treatments is indicated (*P* < 0.05).

At 42 days, T5 (CEO100) maintained the highest BW (2380 g/bird; *P* < 0.01), followed by T2 (EO100; 2288 g/bird), T6 (CEO150; 2218 g/bird), and T1 (EO50; 2212 g/bird). The Control and other groups exhibited significantly lower BW values (2146–2176 g/bird).

Body weight gain (BWG) was significantly affected during all periods (*P* < 0.01). From day 1 to 21, T5 (CEO100) achieved the greatest BWG (706 g/bird), followed by T2 (EO100) and T1 (EO50), which showed higher gains than the Control and other groups. During days 21–42, T5 (CEO100) again had the highest BWG (1630 g/bird), followed by T2 (EO100; 1567 g/bird) and T6 (CEO150; 1558 g/bird). The Control and T3 (EO150) groups had lower gains.

Across the entire experimental period (1–42 days), T5 (CEO100) demonstrated the greatest total BWG (2336 g/bird), followed by T2 (EO100; 2244 g/bird), whereas the Control and T4 (CEO50) recorded the lowest values (2102–2114 g/bird).

Feed intake (FI) was not significantly affected by dietary treatments during any of the experimental periods (*P* > 0.05). Numerically, birds fed 100 mg/kg CEOs (T5; CEO100) showed the highest FI values, while those receiving 50 mg/kg CEOs (T4; CEO50) exhibited the lowest FI during the starter phase (1–21 days). However, these differences did not reach statistical significance during the grower (21–42 days) or overall (1–42 days) periods.

Feed conversion ratio (FCR) was significantly affected across all periods (*P* < 0.05). During the starter phase, T2 (EO100) showed the best FCR (1.43), followed by T5 (CEO100) and T6 (CEO150; both 1.50). The Control, T3 (EO150), and T4 (CEO50) exhibited higher FCRs (1.57–1.59). From 21 to 42 days, T5 (CEO100) and T6 (CEO150) recorded the lowest FCRs (1.40), followed by T4 (CEO50; 1.46) and T2 (EO100; 1.47). Over the total rearing period (1–42 days), T5 (CEO100) maintained the best FCR (1.45; *P* = 0.043), outperforming the Control group (1.56).

### Effect of EO and CEO on the blood biochemistry of heat-stressed broilers

Table 4 presents the effects of dietary supplementation with EOs and CEO on serum glucose, malondialdehyde (MDA), total antioxidant capacity (TAC), and liver function indicators (ALT and AST) in heat-stressed broiler chickens. The results indicated that serum glucose, MDA, ALT, and AST levels were not significantly influenced by the dietary treatments (*P* > 0.05). However, a significant improvement (*P* = 0.003) was observed in serum TAC, where birds fed 100 mg/kg CEO (T5; CEO100) exhibited the highest TAC value (0.80 mM/L), surpassing all other groups. In contrast, the control and other treatments (T1–T4 and T6) showed lower TAC values ranging between 0.45 and 0.62 mM/L. The increase in serum TAC in the T5 group represented approximately a 29% enhancement compared to the control group, indicating that dietary supplementation with CEO100 effectively improved the antioxidant status of broilers under heat stress conditions.

**Table 4.** Effects of dietary essential oils and their encapsulated forms on blood biochemical parameters in heat-stressed broiler chickens

Trait	Experimental groups							MSE	*P*-value
	Control	T1	T2	T3	T4	T5	T6		
**Glucose (mg/dL)**	89.2	65.9	71.5	85.8	88.0	86.6	105.5	21.25	0.128
**AST (U/L)**	136	121	127	133	131	131	116	15.43	0.407
**ALT (U/L)**	16.1	13.9	16.1	15.1	21.8	9.4	11.6	7.03	0.191
**TAC (mM/L)**	0.62^b^	0.58^b^	0.45^b^	0.60^b^	0.48^b^	0.80^a^	0.47^b^	0.14	0.003
**MDA (nmol/ml)**	9.26	14.36	12.46	10.28	10.62	10.50	11.40	3.30	0.291

a,b Means within the same row with no common superscript differ significantly.

T1: 50 mg/kg essential oil; T2: 100 mg/kg essential oil; T3: 150 mg/kg essential oil; T4: 50 mg/kg capsulated essential oil; T5: 100 mg/kg capsulated essential oil; T6: 150 mg/kg capsulated essential oil. MSE: mean square error. Statistical significance among treatments is indicated (*P* < 0.05).

### The effects of essential oils (EO) and encapsulated essential oils (CEO) on hepatic heat shock protein (HSP) expression in heat-stressed broilers

**Figure 2.**
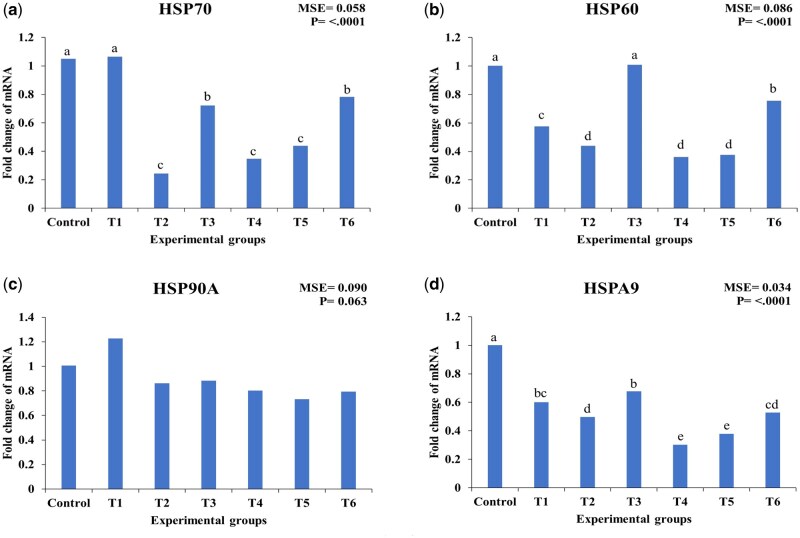
Effect of essential oils (EO) and encapsulated essential oils (CEO) on the expression of hepatic heat shock proteins (HSPs) in heat-stressed broilers. The figure shows (a) HSP70 expression; (b) HSP60 expression; (c) HSP90A expression; (d) HSPA9 expression. T1: 50 mg/kg EO; T2: 100 mg/kg EO; T3: 150 mg/kg EO; T4: 50 mg/kg CEO; T5: 100 mg/kg CEO; T6: 150 mg/kg CEO. MSE: mean square error. Statistical significance among treatments is indicated (*P* < 0.05). Different letters within the same chart indicate significant differences among treatments (a, b, c, d).

As shown in Figure 2, the expression of heat shock proteins in broilers was differentially affected by EO and CEO treatments.

The expression of HSP70 was significantly downregulated by 100 mg/kg EO (T2) and 100 mg/kg CEO (T5), with reductions of 77.1% (*P* < 0.001) and 58.1% (*P* < 0.001), respectively, compared to the control. Treatment with 50 mg/kg CEO (T4) also caused a significant 66.7% decrease (*P* < 0.001). In contrast, 50 mg/kg EO (T1), 150 mg/kg EO (T3), and 150 mg/kg CEO (T6) did not significantly affect HSP70 expression (*P* > 0.05).

HSP60 expression was significantly reduced by 50 mg/kg EO (T1) and 50 mg/kg CEO (T4), with decreases of 42.5% (*P* < 0.01) and 64% (*P* < 0.001), respectively. Similarly, 100 mg/kg EO (T2) and 100 mg/kg CEO (T5) decreased HSP60 expression by 56% and 62.5% (both *P* < 0.001). In contrast, 150 mg/kg EO (T3) resulted in a significant increase in HSP60 expression compared with the control group, whereas 150 mg/kg CEO (T6) did not significantly affect HSP60 levels, as it remained statistically similar to the control (*P* > 0.05).

The expression of HSP90A remained largely unaffected by any treatment (*P* > 0.05). While 50 mg/kg EO (T1) caused a non-significant increase of 21.8%, other treatments, including all CEO doses, resulted in slight but non-significant reductions ranging from 12.9% to 27.7%.

HSPA9 expression was significantly downregulated by 50 mg/kg EO (T1) and 50 mg/kg CEO (T4), with reductions of 40% (*P* < 0.05) and 70% (*P* < 0.001), respectively. Similarly, 100 mg/kg EO (T2) and 100 mg/kg CEO (T5) reduced HSPA9 expression by 50% (*P* < 0.01) and 62% (*P* < 0.001), respectively. Moderate reductions of 32% and 47% were observed with 150 mg/kg EO (T3) and 150 mg/kg CEO (T6), respectively. The reduction in T3 was not significantly different from the control (*P* > 0.05), whereas T6 showed a significant downregulation of HSPA9 expression compared to the control (*P* < 0.05). Additionally, T3 and T6 differed significantly from each other, as indicated by their distinct statistical groupings.

Overall, CEO treatments exhibited stronger inhibitory effects on HSP70, HSP60, and HSPA9 expression than their non-encapsulated EO counterparts at equivalent doses, particularly at 50 mg/kg and 100 mg/kg, whereas HSP90A expression was not significantly affected by any treatment.

### Effect of essential oils and encapsulated essential oils on the expression of hepatic antioxidant enzymes in heat-stressed broilers

**Figure 3.**
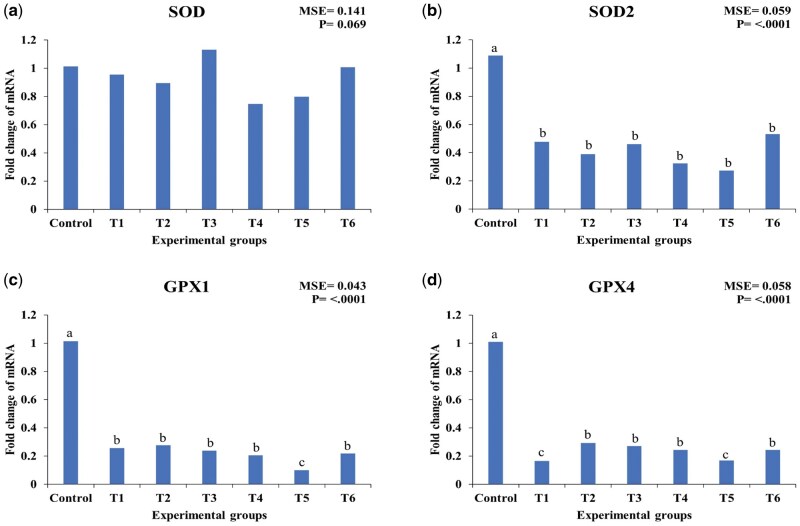
Effect of essential oils and encapsulated essential oils on the expression of hepatic antioxidant enzymes in heat-stressed broilers. The figure shows (a) SOD expression; (b) SOD2expression; (c) GPX1expression; (d) GPX4expression. SOD: superoxide dismutase, SOD2: superoxide dismutase 2, GPX1: glutathione peroxidase 1, GPX4: glutathione peroxidase 4. T1: 50 mg/kg essential oils; T2: 100 mg/kg essential oils; T3: 150 mg/kg essential oils; T4: 50 mg/kg encapsulated essential oils; T5: 100 mg/kg encapsulated essential oils; T6: 150mg/kg encapsulated essential oils. MSE: mean square error. Statistical significance among treatments is indicated (*P* < 0.05). Different letters within the same chart indicate significant differences among treatments (a, b, c).

As illustrated in Figure 3, there were no significant differences in the expression of superoxide dismutase (SOD) among the treatment groups (*P* > 0.05). In contrast, the expressions of superoxide dismutase 2 (SOD2), glutathione peroxidase 1 (GPX1), and glutathione peroxidase 4 (GPX4) were significantly reduced in all treatment groups compared to the control (*P* < 0.05).

Regarding SOD2 expression, supplementation with 50 mg/kg EO (T1) resulted in a 55.96% downregulation (*P* < 0.01), while 100 mg/kg EO (T2) led to a greater reduction of 64.22% (*P* < 0.001). Increasing the dosage to 150 mg/kg EO (T3) resulted in a 57.80% downregulation (*P* < 0.01). Similarly, the CEO groups showed a more pronounced downregulation, with reductions of 70.64% (T4, *P* < 0.001), 75.23% (T5, *P* < 0.001), and 51.38% (T6, *P* < 0.05), respectively.

For GPX1 expression, 50 mg/kg EO (T1) caused a 74.26% downregulation (*P* < 0.001), while supplementation with 100 mg/kg EO (T2) led to a 72.28% reduction (*P* < 0.001). The highest dose, 150 mg/kg EO (T3), downregulated GPX1 expression by 76.24% (*P* < 0.001). Similarly, CEO resulted in a more substantial downregulation, with reductions of 79.21% (T4, *P* < 0.001), 90.10% (T5, *P* < 0.001), and 78.22% (T6, *P* < 0.001) at 50, 100, and 150 mg/kg, respectively.

In the case of GPX4 expression, a significant downregulation was observed across all treatment groups. The administration of 50 mg/kg EO (T1) led to an 83.17% downregulation (*P* < 0.001), while 100 mg/kg EO (T2) and 150 mg/kg EO (T3) reduced expression by 71.29% (*P* < 0.001) and 73.27% (*P* < 0.001), respectively. Similarly, CEO showed a consistent pattern of downregulation, with reductions of 76.24% (T4, *P* < 0.001), 83.17% (T5, *P* < 0.001), and 76.24% (T6, *P* < 0.001) at 50, 100, and 150 mg/kg, respectively.

Among the studied genes, GPX1 was the most affected by the treatments, particularly at 100 mg/kg CEO (T5), which led to a 90.10% downregulation (*P* < 0.001). This suggests that GPX1 is highly responsive to EO and CEO supplementation, with CEO exerting a stronger regulatory effect on its expression.

Overall, the supplementation of EO, particularly in the CEO form, led to a greater downregulation of SOD2, GPX1, and GPX4 compared to EO at the same inclusion levels. The highest reductions were observed with 100 mg/kg CEO (T5), which induced the most pronounced downregulation in SOD2 (75.23%), GPX1 (90.10%), and GPX4 (83.17%), suggesting a stronger effect of CEO in modulating oxidative stress responses in heat-stressed broilers.

### Effects of essential oils and encapsulated essential oils on hepatic antioxidant and stress-responsive gene expression in heat-stressed broilers

**Figure 4.**
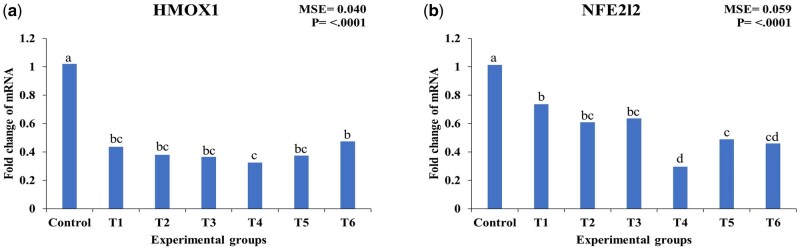
Effect of essential oils and encapsulated essential oils on the expression of hepatic HMOX1 and NFE2L2 in heat-stressed broilers. The figure shows (a) HMOX1 expression; (b) NFE2l2 expression. HMOX1: heme oxygenase 1 gene, NFE2l2: nuclear factor erythroid-derived 2-like 2. T1: 50 mg/kg essential oils; T2: 100 mg/kg essential oils; T3: 150 mg/kg essential oils; T4: 50 mg/kg encapsulated essential oils; T5: 100 mg/kg encapsulated essential oils; T6: 150 mg/kg encapsulated essential oils. MSE: mean square error. Statistical significance among treatments is indicated (*P* < 0.05). Different letters within the same chart indicate significant differences among treatments (a, b, c, d).

Figure 4 shows that HMOX1 expression was significantly lower in all treated groups compared with the control (*P* < 0.05). In the EO groups, HMOX1 expression decreased by 56.86% (T1), 62.74% (T2), and 63.72% (T3). Similarly, CEO treatments resulted in reductions of 68.63% (T4), 62.74% (T5), and 53.92% (T6) compared to the control.

The expression of NFE2L2 was also significantly downregulated across all treatment groups (*P* < 0.05). EO supplementation led to reductions of 26.73% (T1), 39.60% (T2), and 36.63% (T3), while CEO treatments caused more pronounced downregulation, with reductions of 70.30% (T4), 51.49% (T5), and 54.46% (T6) compared to the control. Among the evaluated genes, HMOX1 exhibited the greatest downregulation in response to CEO supplementation, with T4 showing the most substantial reduction (68.63%).

### Effect of EO and CEO on hepatic prooxidant and glucocorticoid receptor gene expression in heat-stressed broilers

**Figure 5.**
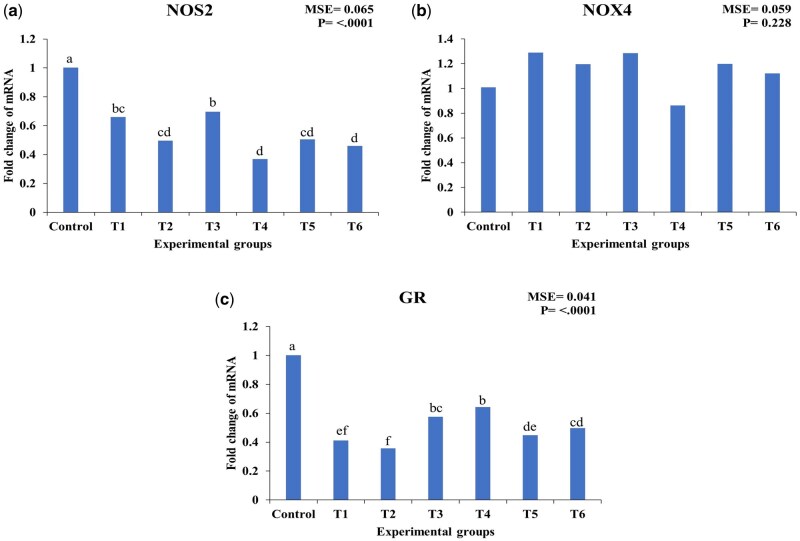
Effect of essential oils and encapsulated essential oils on the expression of hepatic prooxidant and glucocorticoid receptors in heat-stressed broilers. The figure shows (a) NOS2 expression; (b) NOX4 expression; (c) GR expression. NOS2: nitric oxide synthase 2, NOX4: NADPH oxidase 4, GR: glucocorticoid receptor. T1: 50 mg/kg essential oils; T2: 100 mg/kg essential oils; T3: 150 mg/kg essential oils; T4: 50 mg/kg encapsulated essential oils; T5: 100 mg/kg encapsulated essential oils; T6: 150mg/kg encapsulated essential oils. MSE: mean square error. Statistical significance among treatments is indicated (*P* < 0.05). Different letters within the same chart indicate significant differences among treatments (a, b, c, d).

As shown in Figure 5, Dietary supplementation with EO at 50, 100, and 150 mg/kg resulted in a significant downregulation of NOS2 expression by 34%, 50%, and 30%, respectively, compared to the control group (*P* < 0.05). Similarly, CEO supplementation at 50, 100, and 150 mg/kg led to a significant downregulation of NOS2 expression by 63%, 50%, and 54%, respectively, compared to the control (*P* < 0.05). The CEO at 50 mg/kg induced the greatest downregulation of NOS2 among all treatment groups.

NOX4 expression was not significantly affected by dietary supplementation with either EO or CEO at any inclusion level (*P* > 0.05). Although numerical increases and decreases were observed across treatments, these fluctuations did not reach statistical significance and therefore should be interpreted as non-significant biological trends rather than true treatment effects.

EO at 50, 100, and 150 mg/kg significantly downregulated GR expression by 59%, 64%, and 42%, respectively, compared to the control (*P* < 0.05). CEO supplementation at 50, 100, and 150 mg/kg also led to a significant downregulation of GR expression by 36%, 55%, and 50%, respectively (*P* < 0.05). Among all treatments, CEO at 100 mg/kg exhibited the greatest downregulation of GR expression. Among the analyzed genes, GR exhibited the most substantial downregulation across all EO and CEO treatments, particularly with EO at 100 mg/kg showing the greatest reduction of 64% compared to the control.

### Modulation of jejunal glucose transporters by essential oils and encapsulated essential oils in heat-stressed broilers

**Figure 6.**
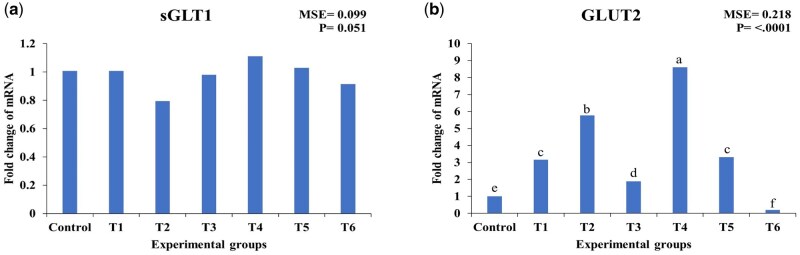
Effect of essential oils and encapsulated essential oils on the expression of jejunal glucose transporters in heat-stressed broilers. The figure shows (a) sGLT1 expression; (b) GLUT2 expression. sGLT1: sodium/glucose cotransporter 1, GLUT2: glucose transporter 2. T1: 50 mg/kg essential oils; T2: 100 mg/kg essential oils; T3: 150 mg/kg essential oils; T4: 50 mg/kg encapsulated essential oils; T5: 100 mg/kg encapsulated essential oils; T6: 150mg/kg encapsulated essential oils. MSE: mean square error. Statistical significance among treatments is indicated (*P* < 0.05). Different letters within the same chart indicate significant differences among treatments (a, b, c, d, e, f).

As shown in Figure 6, the expression of sGLT1 remained unaffected by EO and CEO treatments compared to the control (*P* > 0.05). In contrast, GLUT2 expression was significantly influenced by both EO and CEO supplementation. The highest upregulation was observed in broilers supplemented with 50 mg/kg CEO (T4), showing a 751.5% increase (*P* < 0.05), followed by 100 mg/kg EO (T2) with a 471.3% upregulation (*P* < 0.05). Other treatments also induced upregulation, including 50 mg/kg EO (T1) with 212.9%, 150 mg/kg EO (T3) with 87.1%, and 100 mg/kg CEO (T5) with 227.3% (*P* < 0.05). However, supplementation with 150 mg/kg CEO (T6) resulted in a significant 79.2% downregulation (*P* < 0.05) compared to the control. Among the evaluated glucose transporters, GLUT2 was the most responsive to EO and CEO supplementation

## Discussion

This study highlights the value of using blended EOs and their encapsulated form (CEO) to improve growth and support liver function in broilers exposed to heat stress. The work also explores the physiological and molecular pathways through which these effects occur. Heat stress is a well-recognized challenge in poultry, impairing growth by disrupting metabolism, weakening immune defense, and reducing digestive efficiency(Rostagno, 2020; Brugaletta et al., 2022; Onagbesan et al., 2023). In this study, heat stress conditions were confirmed using the temperature-humidity index (THI), which reached 30.52. This level is classified as moderate to severe heat stress for broilers according to established reference values. (Marai et al., 2001; Hemida et al., 2023; Madkour et al., 2023; Abdel-Fattah et al., 2024). The efficacy of phytogenic feed additives in poultry nutrition remains inconsistent, primarily due to variability in the composition, concentration, and stability of their bioactive constituents (Gadde et al., 2017). Supplementation with the encapsulated EO blend at 100 mg/kg significantly improved broiler growth performance under heat stress. Thymol, eugenol, and carvacrol, major constituents of the EO blend, share structural similarities and have been shown to exert synergistic antimicrobial effects when used in combination at low concentrations (Bassolé and Juliani, 2012). Several reports have documented that dietary blends of thymol, eugenol, carvacrol, and cinnamaldehyde significantly enhance body weight gain and improve feed conversion efficiency in poultry (Liu et al., 2018; Pirgozliev et al., 2019; Irawan et al., 2021; Yang et al., 2021; Gholami-Ahangaran et al., 2022; Noruzi et al., 2022; Elolimy et al., 2025). The observed improvements can be attributed to the beneficial effects of these compounds on gut health and nutrient utilization, which collectively enhance feed intake, body weight gain, feed conversion ratio, and overall digestibility. In addition to their antimicrobial activity, these bioactives exhibit antioxidant, anti-inflammatory, antiviral, and antifungal properties, while also modulating cecal microbiota composition and reinforcing intestinal barrier integrity (Yang et al., 2020; Gholami-Ahangaran et al., 2022; Ibrahim et al., 2022), which may help birds cope with the physiological challenges caused by heat exposure (Kanani et al., 2016; Olfati and Mojtahedin, 2018; Señas-Cuesta et al., 2023; Madkour et al., 2024b; Yilmaz and Gul, 2024). Thymol, carvacrol, and eugenol exhibit antioxidant activity that helps limit oxidative damage, thereby preserving cellular function and energy efficiency in heat-stressed broilers (Surai, 2014; Damasceno et al., 2024; Obianwuna et al., 2024). The growth-promoting effects of EOs are largely associated with enhanced digestive enzyme activity and stabilization of gut microbiota, which together improve nutrient utilization and reduce digestive and metabolic disorders linked to oxidative stress (Kurekci et al., 2014; Ruan et al., 2021; Zhang et al., 2021). The improvements observed in this study are consistent with the previous findings of (Madkour et al., 2024b), who reported that dietary supplementation with 50 mg of oregano, rich in carvacrol (1.69%), and thymol (37.2%), enhanced body weight in heat-stressed broilers. This improvement may be linked to enhanced cellular protection through altered hepatic heat shock protein expression.

Encapsulation plays a key role in improving feed conversion ratio (FCR) by protecting EOs from degradation during feed processing and early digestion, thereby allowing their controlled release and sustained action along the gastrointestinal tract (Hafeez et al., 2016; Nouri, 2019; Moharreri et al., 2021). Previous reports demonstrated that encapsulated oregano (150 mg), cinnamon (200 mg), and clove (50 mg) oils at total doses of 300–400 mg/kg enhanced broiler performance through improved nutrient absorption and stimulation of digestive enzymes including lipase, trypsin, maltase, chymotrypsin, sucrase, and amylase (Meligy et al., 2023). Likewise, microencapsulation of dried kelp with cinnamaldehyde, thymol, and eugenol in a fat matrix improved growth performance at 100 g/ton (day 1–28) and 75 g/ton (day 29–40) (Mullenix et al., 2024).

To investigate the physiological mechanisms underlying these effects, the present study examined hepatic expression of stress- and defense-related genes in heat-stressed broilers, including heat shock proteins (HSP60, HSP70, and HSPA9), antioxidant enzymes (SOD2, GPX1, and GPX4), pro-oxidant markers (NOS2 and NOX4), and the glucocorticoid receptor (GR). Notably, the 100 mg/kg CEO group elicited the strongest transcriptional response, supporting the conclusion that encapsulation enhances the bioavailability and biological activity of EOs under heat stress conditions.

Broilers supplemented with EO or CEO exhibited marked downregulation of HSP60, HSP70, and HSPA9 expression. As these proteins are typically induced during thermal stress to counteract protein damage, their reduced expression indicates that EO supplementation alleviated cellular stress, thereby supporting protein stability and cellular function. These observations are in line with previous reports showing that phytogenic additives can suppress HSP expression in broilers exposed to heat stress.(Akbarian et al., 2014; Greene et al., 2021a; Yilmaz and Gul, 2023; Madkour et al., 2024b), The EO blend’s primary components, cinnamaldehyde, eugenol, and thymol, are known for their antioxidant and anti-inflammatory activities. Although the specific molecular mechanisms remain under investigation, it is likely that these compounds help reduce the formation of reactive oxygen species (ROS), thereby limiting oxidative damage (Goel et al., 2025). One relevant target is nitric oxide synthase (NOS2), which catalyzes nitric oxide production. Nitric oxide readily reacts with superoxide to form peroxynitrite, a damaging oxidant (Lee et al., 2003; Förstermann and Sessa, 2012). The observed reduction in NOS2 expression suggests that EO and CEO supplementation may reduce peroxynitrite-mediated cellular stress, a finding supported by studies linking NOS inhibition to lower inflammation (Bogdan, 2015; Kim and Lee, 2025). In line with this, the current study also documented significant downregulation of SOD2, GPX1, and GPX4, key antioxidant enzymes. While these enzymes typically rise during oxidative stress, their suppression here may indicate that EO and CEO supplementation helped reduce the oxidative load, lessening the need for high endogenous antioxidant activity. This interpretation aligns with prior research showing similar downregulation of hepatic antioxidant genes following supplementation with phytogenics such as rosemary and oregano(Greene et al., 2021b; Madkour et al., 2024a). These effects were attributed to the presence of phenolic compounds like thymol and carvacrol, which contribute directly to redox balance. NRF2, a transcription factor central to oxidative defense, regulates several antioxidant genes, including HMOX1. Under stress, NRF2 activation promotes the expression of detoxification and cytoprotective enzymes. However, both NRF2 and HMOX1 were significantly downregulated in this study, suggesting that EO and CEO supplementation reduced the need for NRF2-driven antioxidant defense. Additionally, since NRF2 also influences lipid and carbohydrate metabolism, the suppression of this pathway may reflect a broader shift in hepatic function toward a more stable metabolic state (He et al., 2020).

Another important stress-related marker, the glucocorticoid receptor (GR), was also downregulated. GR mediates the physiological response to corticosterone, which rises under heat stress. Its reduced expression in supplemented birds implies a dampened stress response, possibly reflecting improved adaptation to the thermal environment. These findings are consistent with prior reports linking GR downregulation to acquired thermotolerance in broilers (Kang et al., 2017; Madkour et al., 2021a).

Serum biochemistry further supported these findings. Glucose, ALT, AST, and MDA levels remained stable across treatments, suggesting that the level of heat stress did not induce hepatic damage. The unchanged glucose levels are notable, as elevated plasma glucose is often seen in heat-stressed birds due to increased hepatic gluconeogenesis (Chand et al., 2018; Qin et al., 2018; Ding et al., 2020; Oladokun and Adewole, 2023), However, this response varies by breed and severity of stress. For instance, some studies found lower glucose levels under HS in certain breeds like Fayoumi, suggesting possible adaptive mechanisms (McCormick et al., 1979; Wang et al., 2018).

Total antioxidant capacity (TAC) was significantly improved in birds receiving 100 mg/kg of CEO, suggesting enhanced systemic antioxidant defense. This is in agreement with other studies where phytogenic formulations elevated serum TAC in broilers under heat stress (Ri et al., 2017; Oladokun et al., 2021; Zhang et al., 2021). The effects of EOs on antioxidant markers remain inconsistent, with some studies reporting no change in TAC, likely reflecting differences in composition, dose, and physiological status of the birds (Su et al., 2021).

In the present work, jejunal glucose transporter expression was also evaluated. While SGLT1 expression remained unchanged, GLUT2 was markedly upregulated in EO- and CEO-treated groups, particularly in the 50 mg/kg CEO group. This aligns with earlier findings that SGLT1 expression may decline or stabilize under prolonged heat stress as an adaptive mechanism to limit glucose uptake (Habashy et al., 2017; Al-Zghoul et al., 2019), In contrast, the upregulation of GLUT2 suggests a compensatory pathway to sustain intestinal glucose absorption during thermal challenge. GLUT2, which mediates the basolateral transport of glucose, galactose, and fructose, proved highly responsive to EO and CEO treatments. The strong increases observed in T4 (751.5%) and T2 (471.3%) indicate that supplementation, particularly in encapsulated form, may counteract the downregulation of GLUT2 reported under chronic heat stress (Habashy et al., 2017; Al-Zghoul et al., 2019), thereby helping to maintain carbohydrate absorption and energy balance. The greater efficacy of CEO at 50 mg/kg compared with non-encapsulated oils likely reflects enhanced bioavailability and targeted intestinal release(Elolimy et al., 2025).Conversely, supplementation at 150 mg/kg CEO (T6) significantly reduced GLUT2 expression, suggesting a biphasic, dose-dependent response consistent with prior reports of inhibitory effects at higher phytochemical concentrations.

Taken together, these results show that GLUT2 is more sensitive than SGLT1 to EO/CEO supplementation under heat stress, underscoring its role in maintaining energy homeostasis. Moderate doses of CEO promoted GLUT2 expression and supported improved glucose utilization, whereas excessive inclusion appeared counterproductive. Although several antioxidant-related genes (such as SOD2, GPX1, GPX4, and HMOX1) exhibited similar trends between EO and CEO at equivalent doses, the overall assessment of growth performance, serum biochemistry, and molecular responses indicates that 100 mg/kg CEO exerted the greatest beneficial effect under heat stress. Broilers receiving this dose showed the most notable improvement in growth performance, particularly in body weight and gain, compared with both the control group and birds receiving the same dose of EO. In addition, serum total antioxidant capacity (TAC) was markedly higher in the 100 mg/kg CEO group than in both the control and the corresponding EO group, reflecting a stronger systemic antioxidant response. These improvements, combined with more consistent modulation of antioxidant-related and cytoprotective genes, support the conclusion that 100 mg/kg CEO provided the most effective physiological and molecular protection under heat-stress conditions.

## Conclusion

Supplementation with blended EOs, particularly at 100 mg/kg CEO, improved growth performance, enhanced antioxidant defenses, and modulated stress-related gene expression in broilers exposed to chronic heat stress. Encapsulation enhanced the stability and utilization of active compounds, leading to reduced expression of oxidative and inflammatory markers, suppression of HSPs (HSP60, HSP70, and HSPA9), and favorable regulation of genes such as NOS2, GR, NRF2, and HMOX1. These findings highlight the potential of CEO as a dietary strategy to improve resilience and performance under thermal stress, while also emphasizing the importance of optimizing dosage to avoid adverse effects. Additionally, CEO increased serum total antioxidant capacity and improved GLUT2 expression, indicating enhanced energy metabolism and intestinal nutrient absorption under heat stress. The biphasic response of GLUT2 at higher CEO doses emphasizes the importance of optimizing supplementation levels. Overall, EO and especially CEO offer promising, natural alternatives to improve broiler health, thermotolerance, and productivity in hot climates through modulation of key physiological and molecular pathways.

## Abbreviations:

ALT: alanine aminotransferase
AST: aspartate aminotransferase
BW: body weight
BWG: body weight gain
cDNA: complementary DNA
CEO: encapsulated essential oil
EOs: essential oils
FCR: feed conversion ratio
GC-MS: gas chromatography-mass spectrometry
GIT: gastrointestinal tract
GLUT2: glucose transporter 2
GPX1: glutathione peroxidase 1
GPX4: glutathione peroxidase 4
GR: glucocorticoid receptor
HMOX1: heme oxygenase 1 gene
HSPs: heat shock proteins
MDA: malondialdehyde
mRNA: messenger RNA
NOS2: nitric oxide synthase 2
NOX4: NADPH oxidase 4
NRF2 or NFE2l2: nuclear factor erythroid 2-related factor 2
qRT-PCR: quantitative real-time PCR
sGLT1: sodium/glucose cotransporter 1
SOD: superoxide dismutase
SOD2: mitochondrial superoxide dismutase
TAC: total antioxidant capacity
THI: temperature-humidity index
